# Replacing Soybean Meal with Distillers Dried Grains with Solubles plus Rumen-Protected Lysine and Methionine: Effects on Growth Performance, Nutrients Digestion, Rumen Fermentation, and Serum Parameters in *Hu* Sheep

**DOI:** 10.3390/ani11082428

**Published:** 2021-08-18

**Authors:** Jiao Chen, Xiaolin Niu, Fei Li, Fadi Li, Long Guo

**Affiliations:** 1 State Key Laboratory of Grassland Agro-Ecosystem, Lanzhou 730020, China; jchen2019@lzu.edu.cn (J.C.); niuxl17@lzu.edu.cn (X.N.); lfei@lzu.edu.cn (F.L.); lifd@lzu.edu.cn (F.L.); 2 College of Pastoral Agriculture Science and Technology, Lanzhou University, Lanzhou 730020, China

**Keywords:** *Hu* sheep, distillers dried grains with solubles, rumen-protected lysine, rumen-protected methionine, growth performance, nutrients digestion

## Abstract

**Simple Summary:**

Improving the economic benefits and precise nutrient supply are hotspots of the sheep breeding industry. Evaluation of the production performance, the rumen fermentation, and blood metabolism indexes found that replacement of soybean meal with distillers dried grains with solubles in a diet with adequate metabolizable protein and amino acids (lysine and methionine) could maintain the normal growth performance of *Hu* sheep. The comprehensive evaluation results provide a reference for reducing production costs, improving production efficiency, and decreasing the nitrogen excretion of the sheep breeding industry. Besides, the study will help in the development of low-protein diets with amino acid balance for sheep.

**Abstract:**

(1) Background: we investigated the influence of dietary soybean meal (SBM) replaced with distillers dried grains with solubles (DDGS) plus rumen-protected (RP) lysine and methionine on the growth performance, nutrients digestion, rumen fermentation, and serum parameters of *Hu* sheep. (2) Methods: ninety *Hu* sheep were allocated to five groups: the control group (CON) which received the SBM diet, the DDGS group (NSM), the DDGS diet with RP lysine group (DRPL), the DDGS diet with RP methionine group (DRPM), and the DDGS diet with a mixture of RP lysine and methionine group (DRPLM). (3) Results: Final BW and carcass weight of the DRPLM and CON groups were greater (*p* ≤ 0.05) compared to NSM, DRPL, and DRPM groups. The DRPLM group tended to increase the dry matter intake (DMI, *p* = 0.06), average daily gain (ADG, *p* = 0.06), dressing percentage (*p* = 0.07), and tail fat weight (*p* = 0.09). The DRPLM group had increased (*p* ≤ 0.05) apparent digestibility and had altered ruminal fermentation characteristics. (4) Conclusions: replacement of SBM with DDGS in a diet with adequate metabolizable protein and by-pass amino acids (lysine and methionine) could maintain the growth performance of *Hu* sheep.

## 1. Introduction

With the continuous increase of feeding costs and the attention of environmental protection issues, the optimal utilization of nutrients has become a top priority. The protein ingredients are the most expensive part of the diet. Inefficient utilization of excess dietary protein in ruminants leads to an unnecessary metabolic burden, environmental pollution, and increased cost of feeding [1]. Therefore, it is necessary to optimize the utilization of protein ingredients for efficient animal production.

Soybean meal (SBM) is the most prominent protein source in the feed industry of China. However, the price of SBM has risen in recent years. Replacing SBM with other low-priced protein feed ingredients is a way to reduce feeding costs, but the balance of nutrients must be considered, especially the amino acids (AAs). Distillers dried grains with solubles (DDGS) is an alternative protein source in diets of animals. As a primary co-product of the corn ethanol industry, DDGS has often been considered a cost-effective source of protein and energy due to its protein and fat content. In the diet of dairy cows, DDGS can be as high as up to 20% of the dietary dry matter (DM) without negatively affecting lactation performance [2], but tends to lack lysine (Lys) [3]. The Lys and methionine (Met) are the major limiting AAs for ruminants in most diets. Balancing growth rations for individual AA is a general method in the breeding industry. Supplementation with rumen-protected (RP) AAs may be a successful strategy for alleviating the possible negative production effects of cheaper, inferior raw materials such as DDGS to replace that of high-quality and expensive feeds. Indeed, many studies have shown small but consistent increases in milk production and dry matter intake (DMI) with dietary addition of rumen-protected amino acids (RPAAs), particularly of RP-Met when AA balance was positive [4].

Due to the excellent prolificacy and growth performance [5], *Hu* sheep have become a significant female parent breed for a domestic animal in China, and the breeding scale of *Hu* sheep has become bigger and bigger in recent years. To further improve the production efficiency and reduce the feeding costs, it is urgent to consider decreasing the cost of protein feeds in the *Hu* sheep industry and promoting the circular economy in the agrifood system, which improves the re-use of agro-industrial byproducts such as DDGS [6]. However, reports of using the DDGS diet in the *Hu* sheep industry are rare. Additionally, a few researchers were studying the application of RPAAs in sheep production. We hypothesized that supplementation of DDGS with RP-Lys + Met could meet or exceed growth performance and carcass traits of sheep fed SBM diets at similar CP levels. The main objectives of the present study were to determine the effects of DDGS supplemented with RP-Lys and RP-Met on growth performance, carcass traits, nutrients digestion, rumen fermentation, and blood biochemistry with a diet at the 14% CP level of *Hu* sheep.

## 2. Materials and Methods

### 2.1. Study Site and Ethical Statement

The study was conducted at the experimental farm of the College of Pastoral Agriculture Science and Technology (Lanzhou University). All experimental protocols were approved by the Animal Care Committee of Lanzhou University (Lanzhou, China), and the experimental procedures used in this study were in accordance with the university’s guidelines for animal research.

### 2.2. Animals, Diets, and Experiment Design

Ninety healthy male *Hu* sheep (body weight, BW; 31.91 ± 2.36 kg) with the age of three months were purchased from a commercial farm and then transported to the experimental farm of the College of Pastoral Agriculture Science and Technology (Lanzhou University). All sheep were injected with the triple and quadruple vaccine as required when they arrived at the farm, and stratified by age and BW to one of 5 treatments, with each treatment having 18 sheep. The treatment groups were: (1) the control group (CON), which received the SBM diet, (2) the DDGS group (NSM), without the SBM and RPAA, (3) the DDGS with RP lysine group (DRPL), (4) the DDGS with RP methionine group (DRPM), and (5) the DDGS with a mixture of RP lysine and methionine group (DRPLM). The addition of Lys and Met in the experimental diet was at amounts of 6 and 2 g/kg, respectively. The four dietary treatments used the DDGS to balance the crude protein (CP) to a similar level as in the CON. The RP-Lys and RP-Met were purchased from Beijing Yahe Nutrition High Tech Co., Ltd. (Beijing, China). The rumen bypass rate of Lys and Met reaches more than 90%. The supplementary amount of RP-Lys and RP-Met and the formulation of diets were referred to as the recommendation of Feeding Standard of Meat-Producing Sheep and Goats of Chinese agricultural industry-standard (NY/T 816-2004) and produced as complete-feed pellets. The ingredients and chemical composition of the diets are presented in Table 1.

This study consisted of an adaptation period of 7 days and the following feeding period of 63 days. At the adaptation period, sheep were fed with the basal diet. Each sheep was housed in a single pen for feeding and watering in a naturally ventilated barn with windows. Sheep received diets twice daily (08:00 and 18:00) to ensure 10% refusal, and had free access to fresh water. The sheep house was regularly disinfected. At the end of the trial, 10 sheep from each dietary treatment group were selected for slaughter to collect carcass traits.

### 2.3. Sample Collection and Performance Measurement

During the feeding trial, the data including BW (determined before the morning feeding) and the quantity of feed offered and refused were recorded for 2 consecutive days, weekly. Daily feed samples were collected and dried at 65 °C for 48 h, and stored at 4 °C until further analysis.

On day 42, 6 sheep were randomly selected from each treatment to measure the dietary apparent digestibility. The method referred to was that of Yang et al. [5]. Briefly, all feces were collected and weighed daily, and then dried at 65 °C for 72 h for further analysis.

Approximately 10 mL of blood samples were collected into a tube using the jugular venous blood sampling at day 42. Serum samples were separated from blood and stored at −20 °C until subsequent analysis of serum parameters, including the concentrations of glucose (GLU), total protein, albumin (ALB), globulin (GLB), alanine aminotransferase (ALT), glutamic oxalacetic transaminase (AST), alkaline phosphatase (ALP), blood urea nitrogen (BUN), creatinine (CR), triglyceride (TG), and total cholesterol (CHO).

Sheep were fasted for 24 h and weighed after 2 h of water deprivation before slaughter. After slaughter (day 63), rumen content pH was determined using a pH meter (PHB-4, Rex, INESA Scientific Instrument CO., LTD, Shanghai, China) and then total rumen (including digesta) was weighted. The rumen content was sampled and stored at −20 °C until subsequent volatile fatty acids (VFAs) and NH3-N analysis. After digesta was removed and washed with sterile PBS (pH 7.0), the empty weights of the rumen were recorded. Subsequently, the weights of the carcass and tail fat were recorded.

### 2.4. Feed and Fecal Sample Analysis

The samples of feed and feces were ground and passed through a 1 mm sieve before analysis of DM (method 924.05; AOAC, 1990), CP (method 988.05; AOAC, 1990), and acid detergent fiber (ADF, method 973.18; AOAC, 1990). The content of neutral detergent fiber (NDF) was determined referring to the Van Soest method.

### 2.5. Blood Analysis

Commercial kits (Nanjing Jiancheng Bioengineering Institute, Nanjing, China; Catalog No. F006-1-1, A045-2-2, A028-2-1, H547-1, C009-2-1, C010-2-1, A059-2-2, C011-2-1, C013-2-1, A110-1-1, A111-1-1) were used to analyze the concentrations of GLU, total protein, ALB, GLB, ALT, AST, ALP, BUN, CR, TG, and total CHO. The Erba XL-640 biochemical analyzer (Erba Lachema s.r.o., Brno, Czech Republic) was used in the subsequent determination.

### 2.6. Rumen VFA Analysis

Approximately 2 g of rumen content was vortexed and washed with 9 mL of sterile PBS (pH 7.0). The liquids were centrifuged at 13,000× *g* at 4 °C for 15 min. The supernatant was removed for VFA analysis, then 20 μL of 85% to 90% orthophosphate acid was added to 1 mL of the VFA sample, and centrifuged again as described above to obtain the final supernatant. VFA contents were determined by methods described by Li et al. [7]. Briefly, a gas chromatograph (TRACE 1300, Thermo Scientific, Milan, Italy) with a DB-FFAP capillary column (DB-FFAP, 30 m × 0.32 mm × 0.25 μm, Agilent Technologies Co., Ltd., Santa Clara, CA, USA) was used. Nitrogen was the carrier, and 2 μL of VFA sample was injected with a syringe, and the temperatures of the injector/detector and the column were 260 and 220 °C, respectively.

### 2.7. Statistical Analyses

A completely randomized design was used to analyze the results. Each animal was viewed as an experimental unit for parameter sweep. The GLM procedure of SAS (SAS Inst. Inc., Cary, NC, USA) was used to perform all statistical analyses.

At each sampled time point, the data for all indexes were pooled and analyzed by a one-way ANOVA. The model of statistics was as below:

Yi = μ + Ai + Bi,

in which Yi, μ, Ai, and Bi represent the dependent variable, overall mean, the diet effect, and the error term, respectively.

Duncan’s test was performed to analyze the multiple comparisons of means. The significant difference was defined as *p* ≤ 0.05 and trends were defined as *p* ≤ 0.10.

## 3. Results

### 3.1. Growth Performance

The growth performance indexes of sheep among different treatments are shown in Table 2. Final BW of the DRPLM group was increased (*p* ≤ 0.05) compared to NSM, DRPL, and DRPM groups, but no difference was observed compared to CON. Compared with other groups, the DRPLM group tended to increase the DMI (*p* = 0.06) and average daily gain (ADG, *p* = 0.06). Initial BW and ratio of feed to gain (F/G) were not significantly different among all treatments (*p* > 0.1).

### 3.2. Carcass Traits

Carcass trait indexes of sheep among different treatments are shown in Table 3. The carcass weight of the DRPLM group was increased (*p* ≤ 0.05) compared to NSM, DRPL, and DRPM groups, but no difference was observed compared to CON (*p* > 0.10). Diet supplemented with RP-AA (Met and Lys) tended to increase the dressing percentage (*p* = 0.07) and tail fat weight (*p* = 0.09). There was no difference in total rumen weight and rumen tissue weight among treatments (*p* > 0.10).

### 3.3. Nutrient Apparent Digestibility

The nutrient apparent digestibility among different treatments is shown in Table 4. The NDF apparent digestibility in the DRPLM group was increased (*p* ≤ 0.05) compared to NSM and DRPM groups, but no difference was observed compared to CON and DRPM groups (*p* > 0.10). Compared with CON and NSM, the DRPLM group tended to increase the apparent digestibility of DM (*p* = 0.07), OM (*p* = 0.07), and ADF (*p* = 0.09).

### 3.4. Rumen Fermentation Parameters

The rumen fermentation parameters among different treatments are shown in Table 5. The ammonia nitrogen concentrations in NSM and AA supplementation groups were decreased (*p* ≤ 0.05) compared to the CON, and the lowest ammonia nitrogen values were obtained in the DRPL group. Compared with the CON or NSM group, the DRPLM group decreased (*p* ≤ 0.05) the butyrate, isobutyrate content, and acetate to propionate ratio. The isovalerate content was greatest in the DRPLM group and least in the DRPM group. The DRPL, DRPM, and DRPLM groups tended to decrease the acetate content (*p* = 0.07) but increased the propionate content (*p* = 0.06) when compared with the CON. The rumen pH, concentration of total VFA, and content of valerate were not different (*p* > 0.10) in all treatments.

### 3.5. Nitrogen Metabolism

The nitrogen metabolism indexes among different treatments are shown in Table 6. The nitrogen apparent digestibility was increased (*p* ≤ 0.05) in DRPML and DRPL groups compared to the CON group. The DRPL group increased (*p* ≤ 0.05) the urine nitrogen content more than CON and DRPM groups. The DRPLM group tended to have decreased nitrogen intake (*p* = 0.07) and fecal nitrogen (*p* = 0.07). The deposition nitrogen and nitrogen retention rate were not significantly different among treatments (*p* > 0.10).

### 3.6. Serum Biochemistry

The serum biochemistry parameters among different treatments are shown in Table 7. The concentration of glucose in the DRPLM group was increased (*p* ≤ 0.05) compared to other groups. The total protein tended to decrease (*p* = 0.09) in the DRPL group compared to the DRPLM and NSM groups, and there was no difference (*p* > 0.10) between CON and DRPL groups. The DRPL and DRPM groups tended to decrease (*p* = 0.09) the ALB concentration compared to other groups. Other indexes of plasma showed no significant differences (*p* > 0.05).

## 4. Discussion

Feeding a diet that provides an adequate AA profile for growth, rather than a high protein level diet in ruminants, is an optimal strategy that could not affect the performance of animals but reduce the elimination of nitrogen and the feed cost. Studies about the effects of supplementation of RPAA on the performance of ruminants are numerous. According to previous studies, responses of DMI to supplemental rumen-protected AA in ruminants were variable. Giallongo et al. reported that a diet supplemented with RP-Met and RP-Lys fed to dairy cows did not affect DMI [8]. Weiss reported that adding RP-Met and RP-Lys to the corn milling products (CMP) diet did not affect the DMI of dairy cows [9]. Chen et al. reported no difference in DMI when early lactating dairy cows were fed a corn silage diet supplemented with methionine analog and lysine-HCl [10]. Li et al. also found that the DDGS supplemented with rumen-protected Lys and Met did not affect the DMI in the lactating cow [11]. In a study by Lee et al. [12], however, Holstein cows fed a diet with RP-Lys and RP-Met tended to increase DMI by 0.7 kg/day compared with the metabolizable protein (MP)-deficient diet group. In the present study, we observed positive effects of supplementation with RP-AA on DMI, ADG, and final BW, which agree with the previous study [13]. However, the discrepancy in DMI responses to the prepartum supply of RPAA among studies is difficult to explain. The supply of an improved AA profile should increase the efficiency of dietary AA utilization for protein synthesis, possibly due to sparing body protein reserves [14]. In the current study, the final BW and carcass weight of *Hu* sheep were not different between DRPLM and CON groups. These results indicated that the DDGS with the rumen-protective lysine and methionine mixture could elicit similar SBM effects on the growth performance and carcass traits. The DMI increased in the DRPLM group and resulted in the ADG tending to increase, which was the main reason for these results. Similar results were observed in two previous experiments in which RP-Lys or RP-Met were added to the diet of Murrah buffaloes [15] or growing Awassi lambs [16]. However, supplementation of individual rumen-protective lysine or methionine decreased the final BW, carcass weight, and tail fat weight, which indicated that the amino acid imbalance could negatively impact the growth performance of cattle [17]. The advantage of improving the balance of absorbable AA is the increased efficiency of the use of absorbed AA for animal body protein synthesis. Therefore, in the next step, the balance of absorbable AA in the diet should be analyzed.

The results of nutrients’ apparent digestibility indicated that the DDGS-supplemented group with mixed RP-Met and Lys could improve the NDF apparent digestibility of *Hu* sheep. The main reason may be that the methionine or its analog could increase the abundance of cellulolytic bacterial representatives in the rumen [18]. The growth of non-cellulosic bacteria could promote the growth of cellulosic bacteria by providing metabolic processes, such as branched-chain fatty acids. Additionally, the rumen bypass rate also affects the nutrient apparent digestibility in the ruminant. Similarly, the lactation response to SBM and RP-Met of corn silage-based diets showed that when RP-Met was added to increase the Lys to Met ratio to 3 to 1, 15% CP was adequate for lactating dairy cows fed corn silage diets supplemented with SBM and lactating approximately 40 kg of milk/day, as well as improved apparent digestibility [19]. However, inconsistent findings were shown in studies of Zhao et al. [20] and Lee et al. [21], who reported that rumen-protected methionine and other essential AAs did not affect the apparent digestibility of dietary nutrients in the dairy cow. The reason was that the different studies used different types of RPAA, while the different RPAAs have different rumen bypass rates, rumen microbial metabolism, and apparent digestibility. Besides, it has been suggested that differences in feed fractional degradation rates in the rumen might be caused by lower rumen ammonia levels in dairy and beef cattle compared with sheep [22].

The concentration of NH3-N mainly reflects the degradation of protein in the rumen. Tamura et al. reported that early lactation cows fed with a diet containing RP-Met did not affect the pH and concentration of ammonia in the rumen [23]. The different results showed that ammonia concentration decreased with the reduced CP content in Holstein cows fed with RP-Met [24]. In the present study, the ammonia concentration of CON was higher than that of other treatments, which indicated that the SBM was not rumen-protected and was rapidly degraded by microbes, therefore it produced more ammonia in the rumen of *Hu* sheep. The rumen pH was not different among all treatments and within a narrower range of 6.0 to 6.5, which indicated that DDGS with RPAA would not affect rumen fermentation status and health. We did not find any differences in total VFA concentrations among treatments, which indicated that replacement of SBM with DDGS supplemented with RPAA may not influence the fermentative capacity of rumen microbes. Similar results were also shown in a previous study [25]. The rate of butyrate and isobutyrate among VFA changed, similar to in the previous study [26]. The variation tendency of propionate concentration and acetate-to-propionate ratio in all DDGS-supplemented groups indicated that the DDGS containing more energy feed from fat changed the VFA production in rumen of *Hu* sheep.

Dietary N input is the primary factor determining metabolism N efficiency, and *Hu* sheep fed the DDGS supplemented with RPLM diets had enhanced N efficiency in the current study [27]. An apparent increase occurred in the relative proportion of fecal N compared with urinary N, while a reduction in apparent nitrogen digestibility occurred in the DRPLM group, similar to previous literature [15,24,28]. The important implication of this shift in the route of N excretion is that manure ammonia emissions will also be expected to decrease because urinary urea is the primary source of ammonium and consequently ammonia in cattle manure [29].

Serum physiological and biochemical indices are usually used to assess the physiological, nutritional, and pathological status of intensively farmed animals. In the present study, dietary supplementation of RP Lys + Met increased the concentrations of serum glucose and tended to increase total protein and ALB concentrations, while the RP-Lys decreased the serum glucose concentration. The literature reported that Met could change key enzymes related to the metabolism of glucose and protein in the liver and facilitate the metabolism of hepatic glucose [30]. Methionine also did not influence glucose metabolism, but it reduced liver lipid accumulation, which promotes hepatic gluconeogenesis and acts to increase concentrations of circulating glucose without changing the utilization of peripheral glucose [31]. Different results have been reported by a previous study investigating the serum biochemical parameters of growing goats, where no differences were observed among the Met- and Lys-supplemented goats [32]. From these data, it is clear that Met has a much greater impact on the concentration of serum glucose and proteins than an equivalent Lys. The dietary inclusion of RPAA resulted in a significant decline in the urea concentration in sheep blood [33]. The reduction in blood urea concentration in the animals fed RPAA might represent an improvement in their nitrogen balance [34]. However, we observed inconsistent results showing that the blood urea did not change significantly, which indicated that *Hu* sheep are in a healthy state after consuming DDGS diets supplemented with RPLM.

## 5. Conclusions

In conclusion, dietary DDGS supplemented with RP-Lys + Met revealed favorable influences on DMI, apparent dry matter digestibility, and nitrogen utilization, and led to increased serum glucose, total protein, and albumin in *Hu* sheep. Therefore, the replacement of SBM with DDGS with RP Lys + Met in diets with adequate metabolizable protein and bypass amino acids (lysine and methionine) could maintain the growth performance of *Hu* sheep.

## Figures and Tables

**Table 1:** Composition and nutrient levels of experimental diets.

Items ^1^	Groups				
	CON	NSM	DRPL	DRPM	DRPLM
Ingredients					
Corn cob	9.00	9.00	9.00	9.00	9.00
Corn stalks	6.00	6.00	6.00	6.00	6.00
Soybean meal	6.00	-	-	-	-
DDGS	-	5.00	5.00	5.00	5.00
Cottonseed meal	6.00	6.00	6.00	6.00	6.00
Corn germ feed	14.40	15.40	15.40	15.40	15.40
Corn bran	10.00	10.00	10.00	10.00	10.00
Concentrate supplement	47.60	47.60	47.60	47.60	47.60
Premix ^2^	1.00	1.00	1.00	1.00	1.00
Total	100.00	100.00	100.00	100.00	100.00
Nutrient level/%DM					
DM	91.07	91.04	91.25	91.11	91.04
CP	13.99	13.87	14.15	14.06	14.80
ME/Mcal·kg^−1^	2.58	2.53	2.53	2.53	2.53
RDP	10.51	9.29	9.35	9.35	9.35
Lys	0.69	0.54	0.96	0.53	0.96
Met	0.27	0.25	0.25	0.39	0.39
NDF	32.61	33.5	31.77	31.70	32.85
ADF	13.83	13.65	13.69	13.26	13.26
Ash	15.31	14.37	14.17	14.58	13.65

^1^ CON = Control; NSM = the DDGS diet without the SBM and RPAA; DRPL = the DDGS diet with RP lysine; DRPM = the DDGS diet with RP methionine; DRPLM = the DDGS diet with a mixture of RP lysine and methionine; DDGS = distillers dried grains with solubles; DM = dry matter; CP = crude protein; RDP = rumen degradable protein. ^2^ Premix provided the following per kg of diets: Fe 3500 mg, Cu 50 mg, Zn 180 mg, Mn 180 mg, Se 3 mg, Co 4 mg, I 3 mg, VA 160,000 IU, VD 350,000 IU, VE 900 IU, VB 1120 mg.

**Table 2:** Effects of dietary supplementation with rumen-protective amino acids on the growth performance of *Hu* sheep.

Items ^1^	Groups					SEM ^2^	*p*-Value
	CON	NSM	DRPL	DRPM	DRPLM		
Initial BW/kg	32.23	31.38	30.89	31.88	32.15	0.27	0.15
Final BW/kg	46.57 ^a,b^	45.88 ^b^	45.40 ^b^	46.19 ^b^	49.03 ^a^	0.44	0.05
DMI (kg·d^−1^)	1.56	1.52	1.50	1.55	1.67	0.02	0.06
ADG (kg·d^−1^)	0.229	0.232	0.232	0.228	0.269	0.01	0.06
F/G	6.99	6.71	6.91	7.04	6.26	0.16	0.19

^1^ CON = Control; NSM = the DDGS diet without the SBM and RPAA; DRPL = the DDGS diet with RP lysine; DRPM = the DDGS diet with RP methionine; DRPLM = the DDGS diet with a mixture of RP lysine and methionine; DMI = dry matter intake; ADG = average daily gain; F/G = gain to feed ratio. ^2^ SEM, standard error of the means. ^a,b^ Means in the same row not bearing a common superscript letter differ (*p* < 0.05).

**Table 3:** Effects of dietary supplementation with rumen-protective amino acids on the carcass traits of *Hu* sheep.

Items ^1^	Groups					SEM ^2^	*p*-Value
	CON	NSM	DRPL	DRPM	DRPLM		
Carcass weight, kg	23.32 ^a,b^	22.97 ^b^	22.98 ^b^	23.13 ^b^	24.47 ^a^	0.18	0.03
Dressing percentage, %	48.54	49.33	49.28	49.33	50.26	0.25	0.07
Total rumen weight, kg	3.58	3.35	3.40	3.58	3.41	0.08	0.48
Rumen tissue weight, kg	0.72	0.70	0.73	0.73	0.79	0.02	0.13
Tail fat weight, kg	1.34	1.15	1.08	1.01	1.35	0.06	0.09

^1^ CON = Control; NSM = the DDGS diet without the SBM and RPAA; DRPL = the DDGS diet with RP lysine; DRPM = the DDGS diet with RP methionine; DRPLM = the DDGS diet with a mixture of RP lysine and methionine. ^2^ SEM, standard error of the means. ^a,b^ Means in the same row not bearing a common superscript letter differ (*p* < 0.05).

**Table 4:** Effects of dietary supplementation with rumen-protective amino acids on nutrient apparent digestibility of *Hu* sheep.

Items ^1^, %	Groups					SEM ^2^	*p*-Value
	CON	NSM	DRPL	DRPM	DRPLM		
DM apparent digestibility	65.00	64.79	65.98	65.25	67.71	0.46	0.07
OM apparent digestibility	67.86	67.36	68.24	67.84	70.39	0.47	0.07
NDF apparent digestibility	37.44 ^a,b^	35.37 ^b^	30.89 ^b^	36.48 ^a,b^	43.91 ^a^	1.28	0.05
ADF apparent digestibility	23.50	21.66	21.61	25.85	26.72	0.84	0.09

^1^ CON = Control; NSM = the DDGS diet without the SBM and RPAA; DRPL = the DDGS diet with RP lysine; DRPM = the DDGS diet with RP methionine; DRPLM = the DDGS diet with a mixture of RP lysine and methionine; DM = dry matter; OM = organic matter; NDF = neutral detergent fiber; ADF = acid detergent fiber. ^2^ SEM, standard error of the means. ^a,b^ Means in the same row not bearing a common superscript letter differ (*p* < 0.05).

**Table 5:** Effects of dietary supplementation with rumen-protective amino acids on the rumen fermentation parameters of *Hu* sheep.

Items ^1^	Groups					SEM ^2^	*p*-Value
	CON	NSM	DRPL	DRPM	DRPLM		
N-NH3 (mg·100 mL^−1^)	27.74 ^a^	14.83 ^b^	10.19 ^b^	15.43 ^b^	11.43 ^b^	1.33	0.00
Rumen pH	6.41	6.25	6.24	6.41	6.30	0.07	0.89
Total VFA (mmol·L^−1^)	54.47	57.89	60.04	52.00	56.21	1.61	0.17
Acetate (%)	45.13	43.78	40.99	41.25	43.43	0.64	0.07
Propionate (%)	36.74	38.08	41.43	43.38	40.76	0.96	0.06
Butyrate (%)	12.81 ^a^	13.71 ^a^	12.90 ^a^	11.39 ^a,b^	9.07 ^b^	0.56	0.02
Isobutytate (%)	0.67 ^a,b^	0.76 ^a^	0.57 ^a,b^	0.49 ^b^	0.50 ^b^	0.04	0.02
Valerate (%)	2.15	2.40	2.53	2.33	2.86	0.13	0.13
Isovalerate (%)	1.06 ^a,b^	0.85 ^a,b^	0.74 ^a,b^	0.66 ^b^	1.33 ^a^	0.09	0.05
Acetate/Propionate	1.26 ^a^	1.13 ^a,b^	0.99 ^b^	0.93 ^b^	1.01 ^b^	0.04	0.03

^1^ CON = Control; NSM = the DDGS diet without the SBM and RPAA; DRPL = the DDGS diet with RP lysine; DRPM = the DDGS diet with RP methionine; DRPLM = the DDGS diet with a mixture of RP lysine and methionine; VFA = volatile fatty acids. ^2^ SEM, standard error of the means. ^a,b^ Means in the same row not bearing a common superscript letter differ (*p* < 0.05).

**Table 6:** Effects of dietary supplementation with rumen-protective amino acids on nitrogen metabolism in *Hu* sheep.

Items ^1^	Groups					SEM ^2^	*p*-Value
	CON	NSM	DRPL	DRPM	DRPLM		
N intake (g·d^−1^)	38.31	37.41	38.54	39.43	29.80	1.46	0.07
Fecal N (g·d^−1^)	12.07	11.66	11.97	11.97	9.06	0.47	0.07
N apparent digestibility %	67.30 ^b^	69.41 ^a,b^	70.55 ^a^	69.43 ^a,b^	71.93 ^a^	0.50	0.03
Urine N (g·d^−1^)	14.97 ^b^	12.34 ^a,b^	15.15 ^a^	10.81 ^b^	12.51 ^a,b^	0.55	0.05
Deposition N (g·d^−1^)	12.79	13.41	10.05	15.93	9.88	1.13	0.16
N retention rate %	30.23	34.96	30.05	39.58	29.62	2.23	0.23

^1^ CON = Control; NSM = the DDGS diet without the SBM and RPAA; DRPL = the DDGS diet with RP lysine; DRPM = the DDGS diet with RP methionine; DRPLM = the DDGS diet with a mixture of RP lysine and methionine; N = nitrogen. ^2^ SEM, standard error of the means. ^a,b^ Means in the same row not bearing a common superscript letter differ (*p* < 0.05).

**Table 7:** Effects of dietary supplementation with rumen-protective amino acids on serum parameters in *Hu* Sheep.

Items ^1^	Groups					SEM ^2^	*p*-Value
	CON	NSM	DRPL	DRPM	DRPLM		
Total Protein/(g·d^−1^)	67.86	69.98	68.06	65.26	70.34	0.83	0.09
ALB (g·L^−1^)	31.8	31.85	30.13	28.88	32.36	0.44	0.09
GLB (g·L^−1^)	35.03	36.80	36.84	33.92	37.97	0.60	0.31
ALB/GLB	0.90	0.86	0.83	0.88	0.85	0.02	0.72
ALT (U·L^−1^)	4.48	6.50	8.54	6.72	4.73	0.59	0.14
AST (U·L^−1^)	110.22	101.80	108.64	105.40	118.00	5.15	0.90
AST/ALT	31.62	17.15	16.86	28.21	30.34	3.78	0.62
ALP (U·L^−1^)	289.45	298.55	296.55	305.50	375.55	14.21	0.29
BUN (mmol·L^−1^)	10.39	9.51	9.35	9.42	9.28	0.17	0.24
CR (μmol·L^−1^)	47.48	47.79	44.75	46.52	44.89	0.88	0.73
GLU (mmol·L^−1^)	2.26 ^b^	2.77 ^a,b^	2.15 ^b^	2.47 ^a,b^	3.22 ^a^	0.12	0.03
TG (mmol·L^−1^)	0.20	0.21	0.19	0.17	0.20	0.01	0.40
Total CHO (mmol·L^−1^)	2.04	1.82	1.74	1.75	1.86	0.05	0.36

^1^ CON = Control; NSM = the DDGS diet without the SBM and RPAA; DRPL = the DDGS diet with RP lysine; DRPM = the DDGS diet with RP methionine; DRPLM = the DDGS diet with a mixture of RP lysine and methionine; GLU = glucose; ALB = albumin; GLB = globulin; ALT = alanine aminotransferase; AST = glutamic oxalacetic transaminase; ALP = alkaline phosphatase; BUN = blood urea nitrogen; CR = creatinine; TG = triglyceride; CHO = cholesterol. ^2^ SEM, standard error of the means. ^a,b^ Means in the same row not bearing a common superscript letter differ (*p* < 0.05).

## Data Availability

The data presented in this study are available on request from the corresponding author.
